# Cost-effectiveness analysis of left atrial appendage occlusion compared with pharmacological strategies for stroke prevention in atrial fibrillation

**DOI:** 10.1186/s12872-016-0351-y

**Published:** 2016-08-31

**Authors:** Vivian Wing-Yan Lee, Ronald Bing-Ching Tsai, Ines Hang-Iao Chow, Bryan Ping-Yen Yan, Mehmet Gungor Kaya, Jai-Wun Park, Yat-Yin Lam

**Affiliations:** 1 School of Pharmacy, Faculty of Medicine, The Chinese University of Hong Kong, 8th Floor, Lo Kwee-Seong Integrated Biomedical Sciences Building, Area 39, Shatin, Hong Kong; 2 Department of Medicine and Therapeutics, Faculty of Medicine, The Chinese University of Hong Kong, Prince of Wales Hospital, Shatin, Hong Kong; 3 Department of Cardiology, Erciyes University School of Medicine, Kayseri, Turkey; 4 Charité University Medicine Berlin, Klinikum Coburg, Coburg, Germany

**Keywords:** Atrial fibrillation, Cost-effectiveness, Left atrial appendage occlusion, Stroke prevention

## Abstract

**Background:**

Transcatheter left atrial appendage occlusion (LAAO) is a promising therapy for stroke prophylaxis in non-valvular atrial fibrillation (NVAF) but its cost-effectiveness remains understudied. This study evaluated the cost-effectiveness of LAAO for stroke prophylaxis in NVAF.

**Methods:**

A Markov decision analytic model was used to compare the cost-effectiveness of LAAO with 7 pharmacological strategies: aspirin alone, clopidogrel plus aspirin, warfarin, dabigatran 110 mg, dabigatran 150 mg, apixaban, and rivaroxaban. Outcome measures included quality-adjusted life years (QALYs), lifetime costs and incremental cost-effectiveness ratios (ICERs). Base-case data were derived from ACTIVE, RE-LY, ARISTOTLE, ROCKET-AF, PROTECT-AF and PREVAIL trials. One-way sensitivity analysis varied by CHADS_2_ score, HAS-BLED score, time horizons, and LAAO costs; and probabilistic sensitivity analysis using 10,000 Monte Carlo simulations was conducted to assess parameter uncertainty.

**Results:**

LAAO was considered cost-effective compared with aspirin, clopidogrel plus aspirin, and warfarin, with ICER of US$5,115, $2,447, and $6,298 per QALY gained, respectively. LAAO was dominant (i.e. less costly but more effective) compared to other strategies. Sensitivity analysis demonstrated favorable ICERs of LAAO against other strategies in varied CHADS_2_ score, HAS-BLED score, time horizons (5 to 15 years) and LAAO costs. LAAO was cost-effective in 86.24 % of 10,000 simulations using a threshold of US$50,000/QALY.

**Conclusions:**

Transcatheter LAAO is cost-effective for prevention of stroke in NVAF compared with 7 pharmacological strategies.

**Condensed abstract:**

The transcatheter left atrial appendage occlusion (LAAO) is considered cost-effective against the standard 7 oral pharmacological strategies including acetylsalicylic acid (ASA) alone, clopidogrel plus ASA, warfarin, dabigatran 110 mg, dabigatran 150 mg, apixaban, and rivaroxaban for stroke prophylaxis in non-valvular atrial fibrillation management.

## Background

Atrial fibrillation (AF) is associated with 4–5 fold increase risk for thromboembolic stroke [1]. Oral anticoagulation therapy with warfarin is the standard therapy for stroke prevention, but is difficult to maintain within the narrow therapeutic range and is under-prescribed in clinical practice. Potential alternatives to warfarin include anti-platelet therapy [2], novel oral anticoagulants (NOACs) such as direct thrombin or factor Xa inhibitors [3, 4] and exclusion of the left atrial appendage (LAA) as a major embolic source [5, 6]. The randomized-controlled WATCHMAN Left Atrial Appendage System for Embolic Protection in Patients with Atrial Fibrillation (PROTECT-AF) trial [5] demonstrated that device occlusion of the LAA orifice by the WATCHMAN device (Boston Scientific, Natick, MA, USA) was non-inferior to warfarin for the prevention of thromboembolic events in NVAF patients. The cost of this device ranges from US$5,770 to US$10,000 depending on the country.

According to recent published economic evaluation studies of LAA compared with warfarin or NOACs, the results indicated that LAA was a cost-effective alternative for stroke prevention in AF patients [7, 8]. However, comprehensive comparison with LAA and each oral anticoagulant should be evaluated to demonstrate significant outcomes. This study estimated the lifetime cost-effectiveness of transcatheter left atrial appendage occlusion (LAAO) for stroke prophylaxis in a hypothetical cohort of 65-year-old patients with non-valvular AF as compared to other pharmacological strategies.

## Methods

### Decision analytical model

A Markov decision analytic model was used to perform a cost-effectiveness analysis from a US healthcare provider perspective expressed in US dollars. The model was developed using TreeAge Pro Suite 2014 software (TreeAge Software, Inc., Williamstown, MA) for evaluating the long-term costs and effectiveness of treatment strategies for stroke prevention. Outcome measures included quality-adjusted life years (QALYs), lifetime costs and incremental cost-effectiveness ratios (ICERs). All costs and QALYs were discounted at an annual rate of 3 %. The ICERs of < US$50,000 per QALY was considered cost-effective [9].

### Model

The model of patients wth AF for stroke prevention was adapted from literature and cardiology consultation [8, 10]. A cohort of 65-year-old patients with non-valvular AF without contraindication to anti-thrombotic therapies was simulated moving between different health states in each Markov cycle of 1 year. The time horizon was lifetime (85 years old). Health states in the model included patient in AF without event, with event before, ischemic stroke (no residual, mild moderate to severe, fatal), transient ischemic attack (TIA), hemorrhage [minor, major, intracranial hemorrhage (ICH), fatal], myocardial infarction (MI), death from vascular cause, and death from all causes. Seven different pharmacological strategies for stroke prevention including acetylsalicyclic acid (ASA) alone (75 to 100 mg), clopidogrel (75 mg) plus ASA, warfarin, dabigatran 110 mg, dabigatran 150 mg, apixaban (5 mg) and rivaroxaban (20 mg) were compared with LAAO. After LAAO, we assumed patients were treated with warfarin for 45 days followed by clopidogrel plus ASA for 180 days, and then lifelong ASA in our study model as in the WATCHMAN trial. There are studies such as the ASA Plavix Feasibility Study with Watchman Left Atrial Appendage Closure Technology (ASAP) study, which used antiplatelet therapy alone after LAAO [5, 11, 12].

### Model parameters

Base-case values for analytic model were derived from published randomized studies including Atrial Fibrillation Clopidogrel Trial with Irbesartan for Prevention of Vascular Events (ACTIVE), Randomized Evaluation of Long-Term Anticoagulation Therapy (RE-LY), Apixaban for Reduction in Stroke and Other Throm-boembolic Events in Atrial Fibrillation (ARISTOTLE), Rivaroxaban Once Daily Oral Direct Factor Xa Inhibition Compared with Vitamin K Antagonism for Prevention of Stroke and Embolism Trial in Atrial Fibrillation (ROCKET-AF), Watchman Left Atrial Appendage System for Embolic Protection in Patients With Atrial Fibrillation (PROTECT-AF), and Prospective Randomized Evaluation of the WATCHMAN LAA Closure Device In Patients with Atrial Fibrillation Versus Long Term Warfarin Therapy (PREVAIL) trials [2–5, 13, 14]. Table 1 summarized the clinical inputs and data sources used in the base-case analysis. Warfarin event rates were pooled warfarin events from RE-LY, ROCKET-AF, and ARISTOTLE trails [3, 4, 13].

**Table 1:** Clinical inputs for base-case value and ranges in decision analytic model

Variable	Base-Case	Range		References
Stroke				
Annual rate of ischemic stroke, %				
Aspirin alone	2 · 80	2 · 80	4 · 50	[2, 18]
Clopidogrel plus aspirin	1 · 90	1 · 69	2 · 11	[2]
Warfarin	1 · 21	1 · 05	1 · 42	[3, 4, 13]
Dabigatran, 110 mg	1 · 34	1 · 31	1 · 55	[3, 15]
Dabigatran, 150 mg	0 · 92	0 · 75	1 · 09	[3, 15]
Apixaban	0 · 97	0 · 78	1 · 19	[13, 16]
Rivaroxaban	1 · 34	1 · 07	1 · 66	[4, 16]
LAA	0 · 84	0 · 40	1 · 10	[5, 14]
Ischemic stroke with clopidogrel plus aspirin or aspirin alone, %				
Fatal (within 30 days)	17 · 90	10 · 10	17 · 90	[17]
Moderate to severe neurologic sequelae	30 · 00	30 · 00	41 · 70	[17]
Mild neurologic sequelae	41 · 00	34 · 80	41 · 00	[17]
No residual neurologic sequelae	11 · 00	11 · 00	13 · 30	[17]
Ischemic stroke with warfarin, dabigatran, apixaban, rivaroxaban or LAA, %				
Fatal (within 30 days)	8 · 20	5 · 50	10 · 90	[15, 17]
Moderate to severe neurologic sequelae	40 · 20	35 · 30	45 · 10	[15, 17]
Mild neurologic sequelae	42 · 50	37 · 60	47 · 40	[15, 17]
No residual neurologic sequelae	9 · 10	6 · 20	12 · 00	[15, 17]
Annual rate of TIA, %	28 · 00	25 · 00	33 · 00	[15, 17]
Hemorrhage				
Annual rate of minor hemorrhage, %				
Aspirin alone	1 · 40	1 · 27	1 · 53	[2]
Clopidogrel plus aspirin	3 · 50	2 · 58	4 · 42	[2]
Warfarin	18 · 63	11 · 40	25 · 80	[3, 4, 13]
Dabigatran, 110 mg	13 · 20	12 · 60	13 · 80	[3, 19]
Dabigatran, 150 mg	14 · 80	14 · 20	15 · 50	[3, 19]
Apixaban	18 · 10	17 · 54	19 · 35	[13]
Rivaroxaban	11 · 80	10 · 94	12 · 88	[4]
LAA (45 days warfarin followed by 180 days clopidogrel and aspirin then lifetime aspirin after LAA)	4 · 28	3 · 70	4 · 86	Assumption
LAA (lifetime aspirin after LAA)	1 · 40	1 · 27	1 · 53	Assumption
Annual rate of major hemorrhage, %				
Aspirin alone	1 · 00	0 · 68	1 · 32	[2]
Clopidogrel plus aspirin	1 · 50	1 · 35	1 · 65	[2]
Warfarin	3 · 32	3 · 09	3 · 57	[3, 4, 13]
Dabigatran, 110 mg	2 · 87	2 · 50	3 · 32	[3, 19]
Dabigatran, 150 mg	3 · 32	2 · 89	3 · 82	[3, 19]
Apixaban	2 · 13	1 · 85	2 · 47	[13]
Rivaroxaban	3 · 60	3 · 06	4 · 08	[4]
LAA (45 days warfarin followed by 180 days clopidogrel and aspirin then lifetime aspirin after LAA)	1 · 54	1 · 30	1 · 77	Assumption
LAA (lifetime aspirin after LAA)	1 · 00	0 · 68	1 · 32	Assumption
Annual rate of ICH, %				
Aspirin alone	0 · 20	0 · 19	0 · 21	[2]
Clopidogrel plus aspirin	0 · 40	0 · 24	0 · 59	[2]
Warfarin	0 · 75	0 · 70	0 · 80	[3, 4, 13]
Dabigatran, 110 mg	0 · 23	0 · 14	0 · 32	[3, 15]
Dabigatran, 150 mg	0 · 30	0 · 20	0 · 40	[3, 15]
Apixaban	0 · 33	0 · 24	0 · 46	[13]
Rivaroxaban	0 · 50	0 · 33	0 · 65	[4]
LAA (45 days warfarin followed by 180 days clopidogrel and aspirin then lifetime aspirin after LAA)	0 · 37	0 · 27	0 · 47	Assumption
LAA (lifetime aspirin after LAA)	0 · 20	0 · 19	0 · 21	Assumption
Annual rate of major hemorrhage as fatal, %				
Aspirin alone	0 · 20	0 · 14	0 · 26	[2]
Clopidogrel plus aspirin	0 · 30	0 · 19	0 · 51	[2]
Warfarin	0 · 90	0 · 50	1 · 80	[3, 4, 13]
Dabigatran, 110 mg	1 · 22	1 · 08	1 · 36	[3]
Dabigatran, 150 mg	1 · 45	1 · 33	1 · 56	[3]
Apixaban	0 · 37	0 · 30	0 · 42	[13]
Rivaroxaban	0 · 20	0 · 16	0 · 40	[4]
LAA (45 days warfarin followed by 180 days clopidogrel and aspirin then lifetime aspirin after LAA)	0 · 45	0 · 38	0 · 51	Assumption
LAA (lifetime aspirin after LAA)	0 · 20	0 · 14	0 · 26	Assumption
Myocardial infarction				
Annual rate of MI, %				
Aspirin alone	0 · 90	0 · 77	1 · 03	[2]
Clopidogrel plus aspirin	0 · 70	0 · 53	0 · 93	[2]
Warfarin	0 · 78	0 · 61	1 · 12	[3, 4, 13]
Dabigatran, 110 mg	0 · 82	0 · 61	1 · 12	[3, 19]
Dabigatran, 150 mg	0 · 81	0 · 60	1 · 09	[3, 19]
Apixaban	0 · 53	0 · 40	0 · 71	[13]
Rivaroxaban	0 · 91	0 · 71	1 · 19	[4]
LAA (45 days warfarin followed by 180 days clopidogrel and aspirin then lifetime aspirin after LAA)	0 · 76	0 · 57	1 · 00	Assumption
LAA (lifetime aspirin after LAA)	0 · 90	0 · 77	1 · 03	Assumption
Pericardial Effusions, %				
Rate of Pericardial effusions				
LAA (within 7 days)	2 · 07	1 · 50	2 · 40	[5, 14]
Success implantation, %				
Rate of LAA device implanted after discontinuing warfarin	0.868	0.8342	0.9018	[18]
Hospitalization				
Annual rate of Hospitalization, %				
Warfarin	20 · 80	15 · 5	26 · 10	[3]
Dabigatran, 110 mg	19 · 40	13 · 49	25 · 32	[3]
Dabigatran, 150 mg	20 · 20	19 · 94	20 · 46	[3]
Apixaban	20 · 80	15 · 50	26 · 10	Assumed equal to Wafarin
Rivaroxaban	20 · 80	15 · 50	26 · 10	Assumed equal to Wafarin
LAA	1 · 08	0 · 00	5 · 00	[5]
Relative Risk of Hospitalization, %				
Warfarin vs. aspirin	1 · 22	0 · 64	2 · 36	[20]
Warfarin vs. clopidogrel plus aspirin	1 · 22	0 · 64	2 · 36	Assumed equal to Aspirin
Death				
Death from vascular cause, %				
Aspirin alone	4 · 70	4 · 48	4 · 92	[2]
Clopidogrel plus aspirin	4 · 70	4 · 18	5 · 26	[2]
Warfarin	2 · 10	1 · 71	2 · 69	[3, 4, 13]
Dabigatran, 110 mg	2 · 43	2 · 23	2 · 63	[3]
Dabigatran, 150 mg	2 · 28	2 · 03	2 · 53	[3]
Apixaban	1 · 80	1 · 54	2 · 10	[13]
All-cause mortality, %				
Aspirin alone	6 · 60	5 · 53	7 · 67	[2]
Clopidogrel plus aspirin	6 · 40	5 · 87	7 · 13	[2]
Warfarin	2 · 89	0 · 50	4 · 13	[3]
Dabigatran, 110 mg	3 · 75	3 · 51	3 · 99	[3]
Dabigatran, 150 mg	3 · 64	3 · 28	4 · 00	[3]
Apixaban	3 · 52	3 · 15	3 · 90	[13]
Rivaroxaban	4.50	4.01	4.99	[4]
LAA	3.20	1.56	4.84	[21]

### Ischemic stroke

The annual ischemic stroke rates were 2 · 8 %, 1 · 9 %, 1 · 21 %, 1 · 34 %, 0 · 92 %, 0 · 97 %, 1 · 34 % and 0 · 84 % for ASA alone, clopidogrel plus ASA, warfarin, dabigatran 110 mg, dabigatran 150 mg, apixaban, rivaroxaban, and LAA occlusion, respectively [2–4, 13, 15–18]. Additionally, TIA accounted for 28 % [15, 17] of all neurological ischemic events in this model. The annual ischemic stroke rate of LAA occlusion was pooled by PROTECT-AF and PREVAIL trails [5, 14]. Proportion of 4 sub-classifications of ischemic stroke (no residual, mild, moderate to severe, fatal) varied according to therapy [15, 17].

### Hemorrhage

Hemorrhages were classified into 4 categories: minor, major, ICH and fatal (Table 1). The annual rates of ICH were 0 · 2 %, 0 · 4 %, 0 · 75 %, 0 · 23 %, 0 · 3 %, 0 · 33 %, and 0 · 5 % for ASA alone, clopidogrel plus ASA, warfarin, dabigatran 110 mg, dabigatran 150 mg, apixaban, and rivaroxaban, respectively [2–4, 13, 15]. The rate of ICH after LAAO was 0 · 37 % for the first year and 0 · 2 % for the second year onwards. A pro-rata method was used to estimate the event rates for LAAO based on patients’ duration of taking ASA, clopidogrel plus ASA, or warfarin therapy (Table 1). We assumed the bleeding rate in the first year after LAAO was lower than warfarin or clopidogrel plus ASA since patients were treated with warfarin for only 45 days followed by clopidogrel plus ASA for 180 days. Bleeding rate from the second year onwards was assumed to be the same as ASA alone [5, 12].

### Myocardial infarction

The annual rates of MI was 0 · 9 % for ASA, 0 · 7 % for clopidogrel plus ASA, 0 · 78 % for warfarin, 0 · 82 % for dabigatran 110 mg, 0 · 81 % for dabigatran 150 mg, 0 · 53 % for apixaban, and 0 · 91 % for rivaroxaban [2–4, 13, 19]. We assumed the rate of MI in the first year after LAAO was lower than warfarin or clopidogrel plus ASA since patients were treated with warfarin for only 45 days followed by clopidogrel plus ASA for 180 days. The rate of MI from the second year onwards was assumed to be the same as ASA alone [5, 12].

### Pericardial effusions

The rate of serious pericardial effusions was 2 · 07 % for patients who received LAAO within 7 days based on the PROTECT-AF and PREVAIL studies [5, 14].

### Success rate of LAA occlusion

LAAO success was defined when anticoagulation could be discontinued after implantation of LAAO device. According to published data, the success rate of LAAO was 86.8 % and others were under warfarin therapy in the LAAO strategy [18].

### Hospitalization

The rates of hospitalization may be occurred after patients with moderate to severe stroke or pericardial effusions which were obtained from the RE-LY [3], PROTECT-AF [5], and the Birmingham Atrial Fibrillation Treatment of the Aged (BAFTA) trial [20]. The hospitalization rates for warfarin, dabigatran 110 mg, dabigatran 150 mg, and LAAO device were 20 · 8 %, 20 · 2 %, 19 · 4 %, and 1 · 08 %, respectively. The rates of apixaban and rivaroxaban were assumed to be the same as warfarin (Table 1).

### Death

The rates of cardiovascular and all-cause mortality for ASA alone, clopidogrel plus ASA, warfarin, dabigatran 110 mg, dabigatran 150 mg and apixaban were 4 · 7 % and 6 · 6 %, 4 · 7 % and 6 · 4 %, 2 · 1 % and 2 · 89 %, 2 · 43 % and 3 · 75 %, 2 · 28 % and 3 · 64 %, 1 · 8 % and 3 · 52 %, respectively [2–4, 13]. The all-cause mortality rates of rivaroxaban and LAAO were 4.5 % and 3.2 % input the model [4, 21].

### Quality of life

Health utilities were obtained from published data (Table 2). The mean utility score was 0 · 998 for ASA, 0 · 987 for warfarin [10]. The utility score for dabigatran of 0 · 994 was based on estimation of previous studies for another direct thrombin inhibitor, ximelagatran [15, 17, 22]. The utility score for dual anti-platelet therapy with clopidogrel plus ASA, and LAAO were assumed to be the same as ASA; otherwise, the utility score for apixaban and rivaroxaban were assumed to be the same as dabigatran in this study.

**Table 2:** Health utilities and costs for base-case value and ranges in decision analytic model

Variable	Base-Case	Range		References
Quality of life				
Mean utility score				
Aspirin alone	0 · 998	0 · 994	1 · 0	[10]
Clopidogrel plus aspirin	0 · 998	0 · 994	1 · 0	Assumed equal to Aspirin
Warfarin	0 · 987	0 · 953	1 · 0	[10]
Dabigatran	0 · 994	0 · 975	1 · 0	[17, 19]
Apixaban	0 · 994	0 · 975	1 · 0	Assumed equal to Dabigatran
Rivaroxaban	0 · 994	0 · 975	1 · 0	Assumed equal to Dabigatran
LAA	0 · 998	0 · 994	1 · 0	Assumed equal to Aspirin
Stroke				
Mild neurologic sequelae	0 · 75	0 · 75	1 · 0	[10]
Moderate to severe neurologic				
sequelae	0 · 39	0 · 39	1 · 0	[10]
Myocardial infarction	0 · 84	0 · 84	1 · 0	[23]
Hemorrhage				
Minor hemorrhage	0 · 8	0 · 5	0 · 99	[15–17, 24]
Major hemorrhage	0 · 8	0 · 5	0 · 99	[15–17, 24]
Cost, US$				
Annual cost of medication or device				
Aspirin alone	10 · 0	5 · 0	15 · 0	[10]
Clopidogrel plus aspirin	1,857 · 0	365 · 0	2,785.5	[10]
Warfarin	180 · 0	60 · 0	270 · 0	[10]
Dabigatran	3,240 · 0	2,500 · 0	4,860	[10]
Apixaban	3,920 · 1	1960 · 1	5,880 · 2	[25]
Rivaroxaban	2,660 · 9	1,330 · 4	3,991 · 3	[25]
LAA	22,500	20,384	24,614	[26], Assumption
Cost of INR + minimal established patient visit	26 · 0	10 · 0	39.0	[10]
Short term cost of neurological event				
Moderate to severe ischemic neurological event	14,680 · 0	6,000 · 0	25,000 · 0	[10]
Minor ischemic neurological event	9,200 · 0	3,500 · 0	15,000 · 0	[10]
TIA	7,500 · 0	3,000 · 0	12,000 · 0	[10]
ICH	38,500 · 0	15,000 · 0	60,000 · 0	[10]
Long term cost of neurological event				
Moderate to severe ischemic neurological event	5,400 · 0	2,000 · 0	8,000 · 0	[10]
Minor ischemic neurological event	2,470 · 0	1,000 · 0	4,000v0	[10]
TIA	5,700 · 0	2,000 · 0	9,000 · 0	[10]
ICH	7,200 · 0	3,000 · 0	12,000 · 0	[10]
Other costs, US$				[10]
Transesophageal echocardiogram	334.0	167.0	501.0	[27]
Major bleeding without residua	4,400 · 0	1,500 · 0	6,000 · 0	[10]
Minor bleeding	69 · 0	34 · 5	200 · 0	[10]
Cost of non-stroke, non-hemorrhage death	10,000 · 0	5,000 · 0	20,000 · 0	[10]
MI	17,000 · 0	5,000 · 0	50,000 · 0	[10]
Hospitalization for stroke	80,964 · 0	40,482 · 0	121,446 · 0	Assumption
Hospitalization for pericardial effusions	73,770 · 0	36,885 · 0	110,655 · 0	Assumption

The mean utility score was 0 · 75 for mild stroke, 0 · 39 for moderate to severe stroke [10]. The utility score of MI (0 · 84) was derived from a nationally representative EQ-5D index scores for a study of chronic conditions in the US [23]. The utility score for minor or major hemorrhage was 0.8 [15–17, 24].

### Cost measurement

Direct inpatient and outpatient medical costs were estimated from a healthcare provider perspective (Table 2). The cost data for the base-case and their ranges were based on a two cost-effectiveness studies of stroke prevention in AF patients [10, 25]. These costs included the costs of anti-thrombotic therapy, hemorrhage, neurological ischemia, dyspepsia, or MI. The estimated cost for LAAO procedure was based on the mean charge of US$14,614 for LAA implantation procedure [26] plus the cost of the LAA occluding device of US$7,885 (US$5,770-US$10,000) that led to the total cost in our analysis as US$22,500. Transesophageal echocardiography (TEE) was performed at the time of LAA device implantation and at 45 days, thus the cost of TEE was US$334 [27].

### Sensitivity analysis

One-way sensitivity analysis was performed by varying CHADS_2_ score, HAS-BLED score, time horizons, and different costs of LAA occlusion for all treatment strategies in this study. The stroke rate for patients with AF was increased by CHADS_2_ score (0–6), which were assumed to be 0 · 8 %, 2 · 2 %, 4 · 5 %, 8 · 6 %, 10 · 9 %, 12.3 % and 13.7 %, respectively [18]. The hemorrhage rates were increased by HAS-BLED score (0–5 score), which were assumed to be 1 · 13 %, 1 · 02 %, 1 · 88 %, 3 · 74 %, 8 · 7 %, and 12 · 5 %, respectively [8]. Time horizon was varied from 20 to 5, 10, and 15 years to assess shorter-term cost-effectiveness from a start-age of 65 years. Sensitivity analysis was also performed with lower and higher costs of LAAO. One-way sensitivity analysis illustrated with tornado diagram was used to assess parameter uncertainty and estimate which parameters had the greatest impact in the model. The parameter was identified as sensitive when either the range was the widest or the ICER value was greater than a threshold of US$50,000. The parameters in warfarin and LAAO strategies were pooled from two or more trials (Tables 1 and 2).

Probabilistic sensitivity analysis (PSA) using 10,000 Monte Carlo simulations was conducted to assess parameter uncertainty. The ranges of all parameters were obtained from published studies and calculating formula of 95 % confidence interval (Tables 1 and 2). A beta distribution was used for those parameters between 0 and 1. Cost data were non-negative quantitative data thus applying a gamma distribution.

## Results

### Base-case analysis

**Fig. 1:**
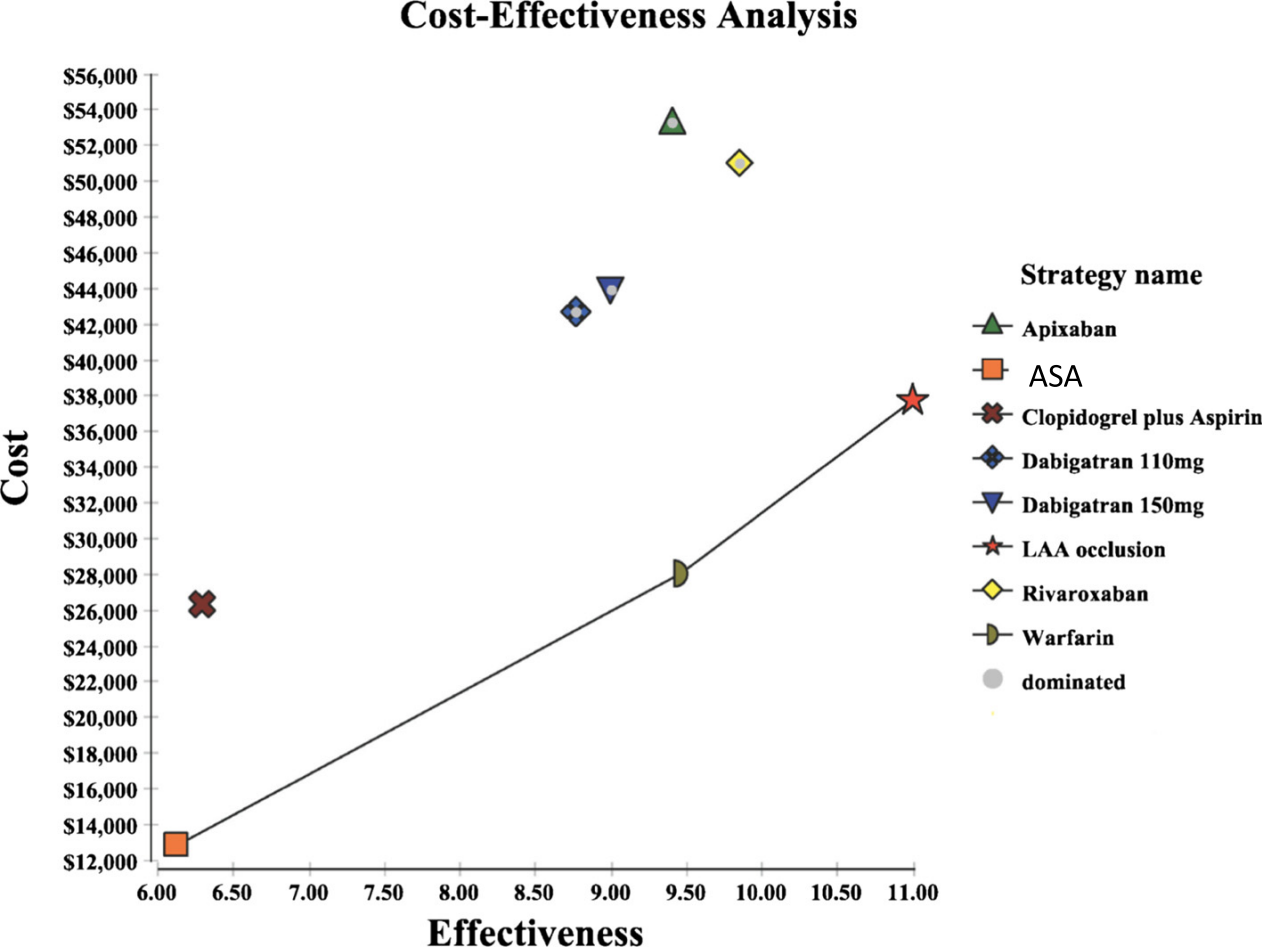
Shows the result of ICER values in comparison of the next-best strategy, and the black line connected from acetylsalicylic acid (ASA) to LAA occlusion as the cost-effectiveness frontier. The effectiveness is defined as the change of quality adjusted life year (QALY) gained. The cost-effectiveness frontier ran from ASA to warfarin to LAA occlusion and its slope increased when moving from the least costly/least effective alternative (ASA) towards the most costly/most effective alternative (LAA occlusion). Clopidogrel plus ASA was an extended dominance* strategy. LAA occlusion is the next more-effective strategy comparing to warfarin, ICER per QALY gained was US$6,298. Dabigatran 110 mg, dabigatran 150 mg, rivaroxaban, and apixaban were dominated by LAA occlusion because those four alternatives were less effective but more costly than LAA occlusion. *Extended dominance: This refers to the observation when the ICER value for a given strategy is higher than that of the next, more effective, alternative. Clopidogrel plus ASA had a higher ICER value than a more effective alternative (warfarin)

Under base-case conditions, LAAO was considered cost-effective compared with the 7 alternative pharmacological stroke prevention strategies for a hypothetical cohort of 65-year-old patient with non-valvular AF (Table 3 and Fig. 1). In descending sequence, the total costs of all strategies were apixaban ($53,315), rivaroxaban ($51,064), dabigatran 150 mg ($43,946), dabigatran 110 mg ($42,712), LAAO ($37,789), warfarin ($28,090), clopidogrel plus ASA ($26,287) and ASA alone ($12,877), respectively. LAAO was associated with the greatest QALYs (10.99 QALYs), followed by rivaroxaban (9.86 QALYs), warfarin (9.45 QALYs), apixaban (9 · 40 QALYs), dabigatran 150 mg (9 · 0 QALYs), dabigatran 110 mg (8.76 QALYs), clopidogrel plus ASA (6 · 29 QALYs) and ASA alone (6 · 12 QALYs).

**Table 3:** Lifetime results of total Costs, total QALYs and ICERs for each stroke prevention strategy (start age at 65-year-old patients)

Therapy	Total Discounted Costs, USD	Total Discounted QALYs, Year	Cost per QALY	ICER, vs. Aspirin	ICER, vs. Clopidogrel plus Aspirin	ICER, vs. Warfarin	ICER, vs. LAA Occlusion	ICER, vs. Dabigatran 110 mg	ICER, vs. Dabigatran 150 mg	ICER, vs. Rivaroxaban	ICER, vs. Apixaban	ICER, vs. Next-best strategy
Aspirin	$12,877	6 · 12	$2,104	---	Dominated^a^	Dominated^a^	Dominated^a^	Dominated^a^	Dominated^a^	Dominated^a^	Dominated^a^	---
Clopidogrel plus aspirin	$26,287	6 · 29	$4,179	$78,882	---	Dominated^a^	Dominated^a^	Dominated^a^	Dominated^a^	Dominated^a^	Dominated^a^	Extended dominance
Warfarin	$28,090	9 · 45	$2,972	$4,568	$571	---	Dominated^a^	Dominated^b^	Dominated^b^	Dominated^a^	Dominated^b^	$571
LAA Occlusion	$37,789	10.99	$3,438	$5,115	$2,447	$6,298	---	Dominated^b^	Dominated^b^	Dominated^b^	Dominated^b^	$6,298
Dabigatran 110 mg	$42,712	8.76	$4,876	$11,301	$6,650	Dominated^c^	Dominated^c^	---	Dominated^a^	Dominated^a^	Dominated^a^	Dominated^a^
Dabigatran 150 mg	$43,946	9.00	$4,883	$10,788	$6,516	Dominated^c^	Dominated^c^	$5,142	---	Dominated^a^	Dominated^a^	Dominated^a^
Rivaroxaban	$51,064	9.86	$5,179	$10,210	$6,940	$56,034	Dominated^c^	$7,593	$8,277	---	Dominated^a^	Dominated^a^
Apixaban	$53,315	9.40	$5,672	$12,329	$8,691	Dominated^c^	Dominated^c^	$16,567	$23,423	Dominated^c^	---	Dominated^a^

*Abbreviations*: *LAA* left atrial appendage, *ICER* incremental cost-effectiveness ratio, *QALY* quality-adjusted life year, *Extended dominance* the alternative has a higher ICER than a more effective comparator

^a^Less costly and less effective strategy

^b^Less costly but more effective strategy

^c^More costly but less effective strategy

The ICER per QALY gained for LAA occlusion compared with ASA alone, clopidogrel plus ASA and warfarin were $5,115, $2,447 and $6,298, respectively. LAAO was dominant (i.e. less costly but more effective) compared to dabigatran 110 mg, dabigatran 150 mg, apixaban, and rivaroxaban.

### Sensitivity analysis

**Fig. 2:**
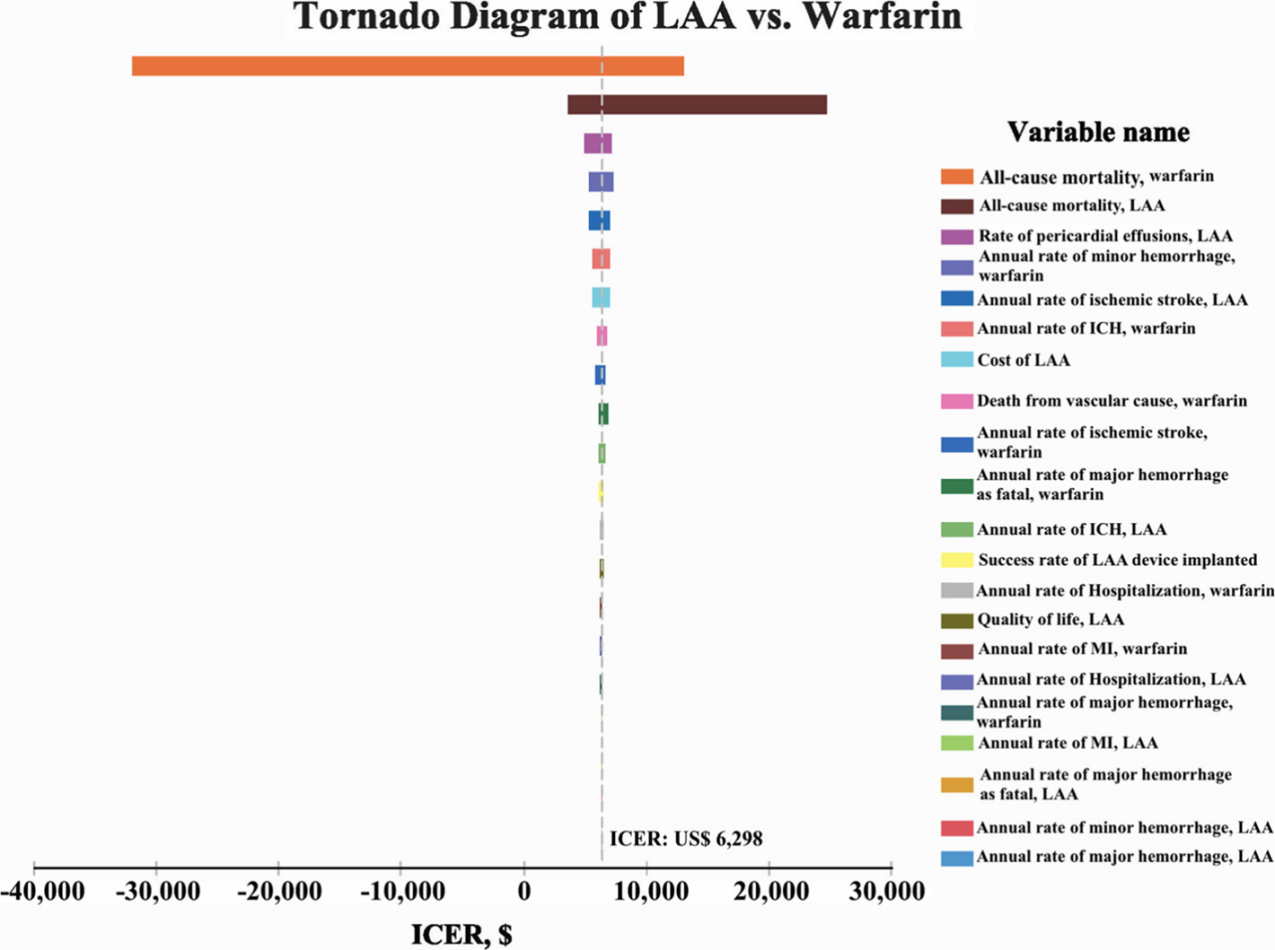
Shows the Tornado diagram with parameters having the greatest impact on the top. The gray dotted line was the ICER value (US$6,298) of LAA occlusion compared to warfarin with base-case result. The all-cause mortalities of warfarin (variable range: 0.5 to 4.13 %) and LAA occlusion (variable range: 1.8 to 2.7 %) had the greatest impact in the model. Even though the range of ICER values of the two parameters were not greater than the threshold of US$50,000, both parameters could still affect the results in the model. The other parameters assessed were not sensitive to the model’s outcomes

**Fig. 3:**
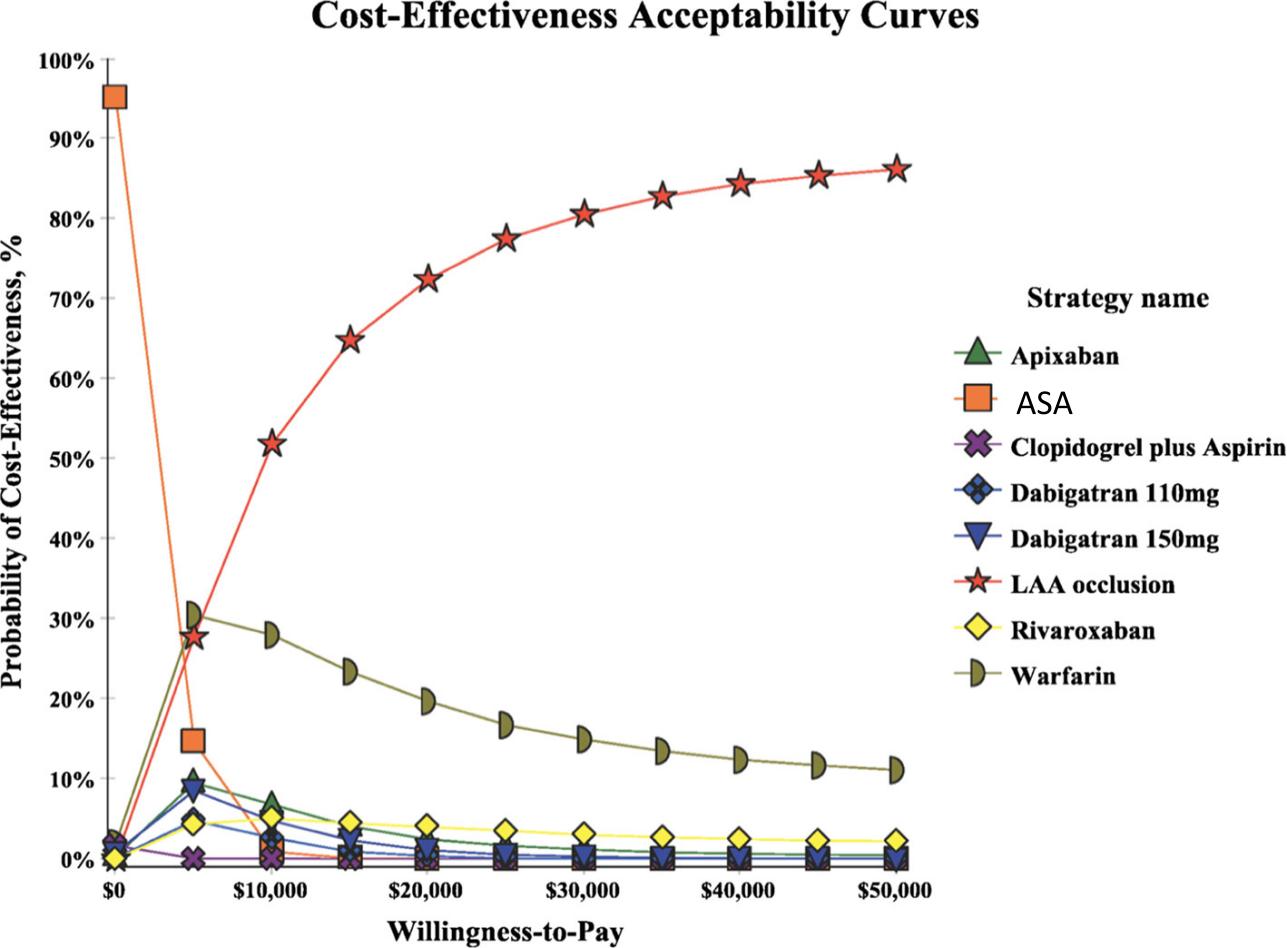
Shows Cost-effectiveness Acceptability Curves (CEACs) for the probability that LAA occlusion strategy was the most cost-effective compared with other 7 strategies for a range of willingness to pay threshold. Given a maximum acceptable ceiling ratio of US$50,000 per QALY gained, the probability of cost-effectiveness for LAA occlusion strategy was 86.24 %

Sensitivity analysis demonstrated that LAAO remained cost-effective compared with other strategies when stroke risk was varied from CHADS_2_ score 0 to 6 (Table 4). In particular, dabigatran 110 mg, dabigatran 150 mg, apixaban and rivaroxaban were dominated by LAAO. When hemorrhage rate was varied by HAS-BLED score from 0 to 5 for anticoagulant drugs in the simulation model, LAAO remained cost-effective compared with each strategy. Varying the time horizon from 20 to 15, 10 and 5 years did not affect the cost-effectiveness of LAAO against all other treatment strategies except for warfarin (ICER: US$74,422) with a short 5 years time horizon. In tornado diagram, the results demonstrated the parameters with greatest impact were all-cause mortality of warfarin (−$32,048–$12,994) and all-cause mortality of LAAO ($3,631–$24,716), respectively (Fig. 2). PSA results demonstrated that the probability of LAAO strategy was the most cost-effective compared with other 7 strategies in 86.24 % of 10,000 Monte Carlo simulations at the threshold of US$50,000/QALY (Fig. 3).

**Table 4:** Sensitivity analysis of total Costs, total QALYs, and ICERs of LAA occlusion compared with each strategy by varying CHADS2 score, HAS-BLED score, time horizons, and LAA occlusion costs

	Aspirin		Clopidogrel + Aspirin		Warfarin		Dabigatran 110 mg		Dabigatran 150 mg		Apixaban		Rivaroxaban		LAAO	
CHADS_2_ Score	Cost	QALY	Cost	QALY	Cost	QALY	Cost	QALY	Cost	QALY	Cost	QALY	Cost	QALY	Cost	QALY
0 (0 · 8 %)	$10,117	6 · 36	$24,998	6 · 42	$27,027	9 · 54	$41,569	8 · 87	$43,685	9 · 02	$52,949	9 · 44	$50,966	9.98	$37,567	11.01
1 (2 · 2 %)	$12,073	6 · 19	$26,627	6 · 25	$30,565	9 · 25	$44,469	8 · 60	$46,627	8 · 75	$55,868	9 · 15	$54,107	9.67	$40,971	10.65
2 (4 · 5 %)	$15,048	5.93	$29,098	5 · 99	$35,853	8 · 81	$48,807	8 · 20	$51,021	8 · 34	$60,207	8 · 71	$58,766	9.19	$46,033	10.11
3 (8 · 6 %)	$19,710	5 · 52	$32,949	5 · 57	$43,894	8 · 11	$55,412	7 · 57	$57,698	7 · 69	$66,749	8 · 01	$65,765	8.44	$53,661	9.25
4 (10 · 9 %)	$22,017	5 · 31	$34,846	5 · 36	$47,753	7 · 76	$58,587	7 · 25	$60,899	7 · 37	$69,859	7 · 67	$69,080	8.07	$57,288	8.83
5 (12.3 %)	$23,327	5.19	$35,919	5.24	$49,905	7.57	$60,359	7.07	$62,684	7.18	$71,586	7.47	$70,915	7.86	$59,299	8.59
6 (13.7 %)	$24,571	5.08	$36,937	5.13	$51,922	7.38	$62,021	6.90	$64,357	7.01	$73,198	7.28	$72,626	7.66	$61,177	8.36
ICER, US$ LAAO vs. each strategy	Score 0: $5,903		Score 0: $2,738		Score 0: $7,170											
	Score 1: $6,479		Score 1: $3,260		Score 1: $7,433											
	Score 2: $7,413		Score 2: $4,110		Score 2: $7,831											
	Score 3: $9,102		Score 3: $5,628		Score 3: $8,568		Dominated		Dominated		Dominated		Dominated		---	
	Score 4: $10,020		Score 4: $6,467		Score 4: $8,911											
	Score 5: $10,580		Score 5: $6,979		Score 5: $9,210											
	Score 6: $11,160		Score 6: $7,505		Score 6: $9,444											
HAS-BLED Score	Cost	QALY	Cost	QALY	Cost	QALY	Cost	QALY	Cost	QALY	Cost	QALY	Cost	QALY	Cost	QALY
0 (1.13 %)	$12,877	6 · 12	$26,287	6 · 29	$36,827	9 · 40	$62,062	8 · 64	$61,719	8 · 88	$72,551	9 · 30	$69,642	9.78	$38,858	10.98
1 (1.08 %)	$12,877	6 · 12	$26,287	6 · 29	$34,333	9 · 42	$59,791	8 · 66	$59,450	8 · 89	$69,999	9 · 31	$66,679	9.79	$38,552	10.99
2 (1.88 %)	$12,877	6 · 12	$26,287	6 · 29	$53,095	9 · 31	$76,902	8 · 55	$76,534	8 · 77	$89,207	9 · 21	$88,969	9.69	$40,851	10.97
3 (3.74 %)	$12,877	6 · 12	$26,287	6 · 29	$88,462	9 · 09	$109,305	8 · 35	$108,814	8 · 55	$125,481	9 · 02	$130,998	9.50	$45,208	10.95
4 (8.7 %)	$12,877	6 · 12	$26,287	6.29	$156,713	8 · 67	$172,530	7 · 94	$171,469	8 · 09	$195,786	8 · 65	$212,146	9.13	$53,713	10.89
5 (12.5 %)	$12,877	6 · 12	$26,287	6.29	$191,794	8 · 45	$205,517	7 · 73	$203,929	7 · 85	$232,133	8 · 45	$253,872	8.94	$58,139	10.87
ICER, US$ LAAO vs. each strategy	Score 0: $5,346		Score 0: $2,680		Score 0: $1,285											
	Score 1: $5,272		Score 1: $2,610		Score 1: $2,687											
	Score 2: $5,768		Score 2: $3,112		Score 2: Dominated											
	Score 3: $6,694		Score 3: $4,060		Score 3: Dominated		Dominated		Dominated		Dominated		Dominated		---	
	Score 4: $8,561		Score 4: $5,962		Score 4: Dominated											
	Score 5: $9,529		Score 5: $6,955		Score 5: Dominated											
Time horizon	Cost	QALY	Cost	QALY	Cost	QALY	Cost	QALY	Cost	QALY	Cost	QALY	Cost	QALY	Cost	QALY
5 years	$6,100	3 · 47	$12,529	3 · 50	$6,898	4 · 02	$17,516	3 · 94	$17,374	3 · 97	$20,356	4 · 03	$17,040	4.08	$24,015	4.25
10 years	$9,788	5 · 06	$19,929	5 · 15	$14,876	6 · 63	$29,508	6 · 36	$29,707	6 · 46	$35,233	6 · 63	$31,205	6.81	$29,026	7.28
15 years	$11,801	5 · 78	$24,048	5 · 92	$22,111	8 · 34	$37,471	7 · 85	$38,190	8 · 02	$45,834	8 · 31	$42,397	8.63	$33,699	9.44
ICER, US$ LAAO vs. each strategy	0 5 years: $22,968		0 5 years: $15,315		5 years: $74,422		5 years: $20,965		05 years: $23,718		5 years: $16,632		5 years: $41,029			
	10 years: $8,666		10 years: $4,217		10 years: $21,769		10 years: Dominated		10 years: Dominated		10 years: Dominated		10 years: Dominated		---	
	15 years: $5,983		15 years: $2,742		15 years: $10,535		15 years: Dominated		15 years: Dominated		15 years: Dominated		15 years: Dominated			
LAAO costs	Cost	QALY	Cost	QALY	Cost	QALY	Cost	QALY	Cost	QALY	Cost	QALY	Cost	QALY	Cost	QALY
Low cost ($20,384)	$12,877	6.12	$26,287	6.29	$28,090	9.45	$42,712	8.76	$43,946	9.00	$53,315	9.40	$51,064	9.86	$36,731	10.99
Base-case ($22,500)	$12,877	6.12	$26,287	6.29	$28,090	9.45	$42,712	8.76	$43,946	9.00	$53,315	9.40	$51,064	9.86	$37,789	10.99
High cost ($24,614)	$12,877	6.12	$26,287	6.29	$28,090	9.45	$42,712	8.76	$43,946	9.00	$53,315	9.40	$51,064	9.86	$38,846	10.99
ICER, US$ LAAO vs. each strategy	Low cost: $4,898		Low cost: $2,222		Low cost: $5,611											
	Base-case: $5,115		Base-case: $2,447		Base-case: $6,298		Dominated		Dominated		Dominated		Dominated		---	
	High cost: $5,332		High cost: $2,672		High cost: $6,984											

*Abbreviations*: *LAAO* left atrial appendage occlusion, *ICER* incremental cost-effectiveness ratio, *QALY* quality-adjusted life year, *CHADS*_*2*_ congestive heart failure, hypertension, age > 75, diabetes mellitus, and previous stroke/transient ischemic attack, *HAS*-*BLED* hypertension, abnormal renal/liver function, stroke, bleeding history or predisposition, Labile international normalized ratio, Elderly (>65 years), drugs/alcohol concomitantly, *ICER* calculated as the difference in cost divided by the difference in QALYs for each therapy compared with LAAO strategy, *Dominated* LAAO is less costly but more effective strategy compare with each strategy

## Discussion

Previous study has demonstrated the cost-effectiveness of LAAO, dabigatran and warfarin in the management of NVAF [28]. This was the first comprehensive analysis to compare the cost-effectiveness between seven pharmacological strategies including newer oral anticoagulants and transcatheter LAA occlusion for stroke prevention in NVAF patients. We demonstrated that LAAO was associated with the highest QALYs gained and the lowest ICER per QALY gained compared to 7 other pharmacological regimens in the prevention of AF-related stroke. Sensitivity analysis also demonstrated that LAAO remained cost-effective compared with all 7 alternative strategies across the spectrum of stroke risks, bleeding risk and time horizon.

Atrial fibrillation is a growing problem in an aging society. It causes >50 000 strokes and $12 billion in medical expenditure each year in United States. Warfarin used to be the standard of care in preventing stroke but it is difficult to be used conveniently and safely [29]. NOACs may be comparable to warfarin in terms of clinical efficacy but the benefit does not come without risk of bleeding. Transcatheter LAAO potentially reduces both risks of stroke and bleeding associated with long-term anticoagulation and the 2012 European Society of Cardiology Guidelines recommended such intervention can be considered in patients with high stroke risk and contraindications for oral anticoagulants [30]. A few studies attempted to evaluate the cost-effectiveness of these newer stroke preventive strategies. One key analysis based on the RE-LY study [3] showed the ICERs of dabigatran 110 mg and 150 mg compared with warfarin were US$16,147–115,129 and US$39,680–263,543, respectively, which were much higher compared to the ICER for LAAO in our current study. SM Singh, A Micieli and HC Wijeysundera [8] demonstrated LAAO was cost-effective as compared to dabigatran and warfarin but they did not address the impact of other commonly used NOACs and the treatment duration on the cost-effective performance of the device therapy [28]. In current analysis, we demonstrated the superior cost-effectiveness of the device compared to other NOACs, which is independent of stroke risk (CHADS_2_ score), bleeding risk (HAS-BLED score) and treatment duration (i.e. device strategy was cost-effective even at 5 year follow-up). In particular, LAAO was considered cost-effective comparing to all alternative strategies when HAS-BLED score and CHADS_2_ score were varied. Considering most adverse events occur during and shortly after device implantation [5, 20], while events with oral anticoagulants develop continuously over time, our findings may provide additional insights in selecting specific therapy for individual patient groups.

Three endovascular LAA occluding devices have been widely used in humans and many other new devices are under pre-clinical evaluation [31]. The PLAATO device was the oldest with reported favorable clinical results up to 5 years but the device has been withdrawn from the market because of financial considerations [32]. PROTECT-AF trial [5] showed the WATCHMAN device was non-inferior to warfarin in reducing ischemic stroke in AF patients with CHADS_2_ score of ≥1 and the device arm was associated with less hemorrhagic stroke. Early registry results with Amplatzer Cardiac Plug (St Jude Medical Inc, US), consistently reported a high implantation success rate >95 %, implying its wide applicability to AF patients [6, 33]. The longest follow-up data were also shown to demonstrate the promising results with Amplatzer device in AF patients for stroke prevention [33]. While the device therapy addresses both the concerns of inconvenience (no issue with drug interaction, blood monitoring and compliance) and safety (bleeding) associated with long term oral anticoagulant usage, it also has shortcomings in particular procedural-related complications [5, 6, 12, 14] and the risks of having incomplete LAAO and thrombus formation on the device during long-term follow up. The costs of managing these events needed to be studied especially when the device strategy has been widely adopted in in-experienced centers.

### Limitations

There are a number of limitations of the current study. Firstly, there was no directly comparative trial between LAAO and oral anticoagulation. Secondly, the base-case values of the current model simulation were derived from individual clinical trials from different countries and healthcare systems with variable costs of management. Thirdly, a number of base-case assumptions were necessary when trial data were lacking. Fourthly, data from randomized clinical trials could not be generalizable to “real world” clinical practice. It should also be noted that only direct medical cost was considered in the analysis. Fifthly, we assumed that warfarin was discontinued after 45 days post LAAO although some patients may require warfarin beyond 45 days when TEE confirmed clots or device leak. Furthermore, the long-term follow-up data for the newer LAAO devices were obtained from a single study with 10-year follow up of Amplatzer left atrial appendage occlusion [33], it may add to model uncertainties and parameter uncertainties in the results, however, sensitivity analyses demonstrated the robustness of study results.

## Conclusions

In conclusion, our Markov analytic model demonstrated that transcatheter LAAO was cost-effective compared to ASA alone, clopidogrel plus ASA, warfarin, dabigatran 110 mg, dabigatran 150 mg, rivaroxaban and apixaban for stroke prevention in patients with NVAF.

## Abbreviations

AF: Atrial fibrillation
ASA: Acetylsalicyclic acid
ICERs: Incremental cost-effectiveness ratios
ICH: Intracranial hemorrhage
LAA: Left atrial appendage
LAAO: Left atrial appendage occlusion
NOACs: Novel oral anticoagulants
NVAF: Non-valvular atrial fibrillation
QALYs: Quality-adjusted life years
TEE: Transesophageal echocardiography
TIA: Transient ischemic attack
